# Updates and Outlook for GAIMH

**DOI:** 10.1177/27536130251347754

**Published:** 2025-06-10

**Authors:** Erik J. Groessl, Frederick M. Hecht

**Affiliations:** 1 Herbert Wertheim School of Public Health, University of California San Diego, Los Jolla, CA, USA; 2 School of Medicine, University of California, San Francisco, CA, USA

Having returned from the recent ICIMH meeting in Seattle, WA, we are feeling inspired and hopeful about our journal’s mission to advance whole person health through the dissemination of evidence and knowledge about integrative medicine and health. Despite many concerns about research funding, misinformation, and academic pursuits more generally, we would like to take this opportunity to focus on some known positives and update our readers and Consortium members on progress at GAIMH.

GAIMH (formerly Global Advances in Health and Medicine) has been the official journal of the Academic Consortium of Integrative Medicine and Health (ACIMH) since 2017. Over that time, there has been steady growth in the number of manuscripts submitted and the number of full-text published manuscripts that have been downloaded. In 2024, 95 manuscripts were submitted and about 230,000 manuscript downloads occurred. Most importantly, after a required 2-year waiting period, our resubmitted application to be indexed in the Emerging Sources Citation Index (ESCI) and to receive an impact factor was approved. Currently, citations of our manuscripts published during 2023 and 2024 are being tracked in 2025 and will result in an impact factor in 2026. Until then, we will continue our efforts to strengthen the journal, reduce the time to first full decision below 60 days, and to provide prompt responses and service to author inquiries.

We have recently concluded three Special Collections,

- Remote Delivery of Mindful Movement Interventions,
- Nature and Integrative Health,
- The Science of Integrative Health Equity.

All of the manuscripts in these successful collections (and in previous collections) are available for download on our website, although a few more revised manuscripts may still be in process. https://journals.sagepub.com/collection-index/gam

For 2025, we have recently opened a number of new Special Collections for submission (https://journals.sagepub.com/page/gam/collections/special-collections/index)

- Therapeutic Use of the Arts in Clinical and Community Settings
- Advancing Research Methodology in Integrative Medicine and Health
- Teaching Kitchens, Nutrition Education, and the Future of Food as Medicine in Clinical and Community Health

In addition to our special collections, we plan to continue several other special initiatives. We occasionally publish clinical impact reviews of Cochrane Reviews on relevant topics. Our first manuscript in this series looked at the implications of a Cochrane Review of Acupuncture for Chronic Low Back Pain. Currently, we are planning the next clinical impact review focused on a recent Cochrane Review of probiotics for management of functional abdominal pain disorders in children

We also have assisted in the dissemination of research reporting guidelines by publishing relevant guidelines as a secondary journal of publication. We have done this for biofield research reporting guidelines in 2023 and are planning to do this for music therapy research reporting guidelines in the coming month or two.

As an open access journal, we realize that authors have many choices for journals to which they could submit their work. Thus, we continue to strive to keep our publishing fees as low as possible and would like to share information on a variety of discounts that are available to Corresponding Authors who are affiliated with institutions in countries, areas and territories in Research4Life’s Group A list(100% waiver) or Research4Life’s Group B list (50% waiver).

There are also many institutions participating in Open Access Agreements at Sage that receive major publishing fee discounts. Last, but not least, we continue to offer a 20% discount for Corresponding Authors who are ACIMH members.

As Co-Editors-in-Chief, we are committed to continuous improvement efforts at GAIMH. One area we can use assistance with is identification of qualified peer-reviewers for our manuscripts. If you are willing to participate as a peer-reviewer, please complete a profile here if you have not already; your help is greatly appreciated.

We welcome your feedback and perspectives as researchers, educators, and clinicians in integrative medicine, and we hope you will consider GAIMH as a home for your high-quality manuscripts. We invite submissions across the research spectrum within integrative medicine and health; from basic science and translational to clinical, pilot feasibility to mechanistic to implementation. We remain committed to being a leading platform for dissemination of rigorous evidence-based research and clinical innovation that advances scientific knowledge and helps shape the vision of the field of integrative medicine and whole person health.

